# Phenolic Profile and Antioxidant Capacity of Extracts from Papache (*Randia echinocarpa* Moc. & Sessé Ex DC), a Plant Used in Traditional Mexican Medicine

**DOI:** 10.3390/plants15071086

**Published:** 2026-04-01

**Authors:** Refugio Riquelmer Lugo-Gamboa, Norma Patricia Muñoz-Sevilla, Juan Pablo Apún-Molina, Jesús Arturo Fierro-Coronado, Abraham Cruz-Mendívil, Mauro Espinoza-Ortiz, Maribel Valdez-Morales, Apolinar Santamaria-Miranda

**Affiliations:** 1 Instituto Politécnico Nacional-CIIDIR Sinaloa, Blvd. Juan de Dios Bátiz Paredes #250, Guasave 81049, Sinaloa, Mexico; rlugog2115@alumno.ipn.mx (R.R.L.-G.); japun@ipn.mx (J.P.A.-M.); jfierroc@ipn.mx (J.A.F.-C.); mespinozao1700@alumno.ipn.mx (M.E.-O.); mvaldezmo@ipn.mx (M.V.-M.); 2 Instituto Politécnico Nacional-CIIEMAD, 30 de junio de 1520 s/n, La Laguna Ticoman, Gustavo A. Madero, Ciudad de Mexico 07340, Mexico; nmunozs@ipn.mx; 3 SECIHTI-Instituto Politécnico Nacional, CIIDIR Sinaloa, Blvd. Juan de Dios Bátiz Paredes #250, Guasave 81049, Sinaloa, Mexico; acruzm@secihti.mx

**Keywords:** polyphenols, antioxidant capacity, medicinal plant

## Abstract

*Randia echinocarpa* is an endemic shrub species of Mexico, commonly known as papache in the state of Sinaloa, where it has traditionally been used in medicinal practices. The present study evaluated the phenolic profile and antioxidant capacity of different tissues (leaf, bark, and fruit pulp) of *R. echinocarpa*. Phenolic compounds were characterized using HPLC–PDA–MS, which allowed the identification of seven compounds in the leaf, six in the bark, and six in the fruit pulp. Chlorogenic acid, ellagic acid, and rutin were among the most abundant compounds detected. Total phenolic content varied depending on tissue and season, with the highest concentration observed in leaves during autumn (2.770 ± 0.011 mg GAE g^−1^) and the lowest in bark during winter (0.437 ± 0.009 mg GAE g^−1^). This study also reports, for the first time, the concentrations of tannins and flavonoids in *R. echinocarpa*, with the highest content found in leaves during autumn (0.261 ± 0.003 mg EE g^−1^ and 2.186 ± 0.005 mg RE g^−1^, respectively). Antioxidant capacity was evaluated using DPPH and ABTS radical scavenging assays, with leaf extracts showing the highest activity, with IC_50_ values of 0.82 mg mL^−1^ and 1.21 mg mL^−1^, respectively. These results provide new information on the phenolic composition and antioxidant potential of *R. echinocarpa*, contributing to the phytochemical characterization of this traditionally used medicinal species.

## 1. Introduction

Mexico has a vast herbal and ethnobotanical heritage and is recognized as one of the most biodiverse countries in the world, harboring approximately 10–12% of all terrestrial species. It ranks fifth globally in plant biodiversity, with nearly 10% of the world’s flora reported by 2015. Of these species, approximately 4000 have been identified as having therapeutic potential [1,2,3]. However, it is estimated that only about 5% of these species have undergone chemical, pharmacological, or biomedical validation in Mexico [3].

*Randia echinocarpa* Moc. & Sessé ex DC. is an endemic shrub species of Mexico belonging to the Rubiaceae family. It is distributed along the Pacific coast in conserved tropical deciduous forests [4]. Commonly known as papache in the state of Sinaloa, this plant produces an irregularly structured fruit with edible pulp that has been traditionally used to treat various ailments, including cancer, malaria, diabetes, and kidney, lung, circulatory, and gastrointestinal disorders [4,5]. Different tissues of the plant are harvested at specific times of the year according to traditional medicinal practices: the leaves can be collected year-round, the bark during spring–summer, and the fruit pulp during autumn–winter. In addition, the species is recognized as an important melliferous plant [6].

Previous studies have reported several biological activities associated with *R. echinocarpa* [7]. Subsequent studies identified soluble melanins in the fruit with inhibitory activity against α-glucosidase, suggesting potential applications in the management of type II diabetes [8]. These soluble melanins have also demonstrated in vivo antioxidant and immunomodulatory activities and have been reported as non-toxic, suggesting their potential use as bioactive ingredients in functional foods, supplements, and phytotherapeutic formulations [9]. Additionally, methanolic extracts from seedlings and callus tissues have shown free radical scavenging activity against DPPH and ABTS radicals [9]. Many of these biological activities are commonly associated with the presence of phenolic compounds, which are widely recognized for their antioxidant properties due to their ability to neutralize free radicals and reduce oxidative stress [10].

Recent studies have identified several phenolic compounds in *R. echinocarpa* biomass and culture supernatants, including chlorogenic and salicylic acids as major constituents [11]. However, information on the phenolic composition of different tissues of *R. echinocarpa* and the influence of seasonal variation on the accumulation of these compounds remains limited. Furthermore, post-harvest processing methods such as drying may influence the stability and preservation of phenolic compounds.

Therefore, the aim of this study was to characterize the phenolic profile of different tissues (leaf, bark, and fruit pulp) of *R. echinocarpa* using HPLC–PDA–MS, evaluate their antioxidant capacity, and assess how drying methods and seasonal collection influence phenolic preservation.

## 2. Results

### 2.1. Total Phenolic Content (TPC)

Total phenolic content was determined in ethanolic extracts of fruit pulp, leaves, and bark of *R. echinocarpa* collected throughout the year and subjected to three drying methods: freeze drying (0.045–0.090 mbar, −85 °C) and oven drying at 45 °C and 75 °C.

The highest TPC was recorded in leaves collected during autumn and dried at 45 °C (2.770 ± 0.011 mg GAE g^−1^), followed by lyophilized leaves collected during autumn (2.617 ± 0.048 mg GAE g^−1^). In contrast, the lowest value was observed in bark collected during winter and oven-dried at 45 °C (0.437 ± 0.009 mg GAE g^−1^), representing an approximately six-fold difference between the highest and lowest values. These differences were statistically significant among tissues and seasons (Table 1; *p* < 0.05), indicating that both plant tissue and season influence phenolic accumulation in *R. echinocarpa*.

Across drying treatments, oven drying at 45 °C produced comparable or slightly higher phenolic concentrations than freeze drying in some samples, particularly in autumn leaves. This may be related to improved phenolic extractability due to partial disruption of plant tissues during moderate thermal drying. In contrast, drying at 75 °C generally resulted in lower phenolic concentrations, possibly due to degradation of thermolabile compounds. The season also influenced phenolic accumulation, with leaves collected during autumn consistently showing higher phenolic levels compared with other seasons (Table 1). This pattern may reflect seasonal changes in plant metabolism and the biosynthesis of phenolic compounds involved in plant defense responses.

### 2.2. Total Flavonoid Content (TFC)

Total flavonoid content was determined in ethanolic extracts of fruit pulp, leaves, and bark of *R. echinocarpa* collected throughout the year and subjected to three drying methods: freeze drying (0.045–0.090 mbar, −85 °C) and oven drying at 45 °C and 75 °C. The highest TFC was recorded in leaves collected during autumn and dried at 45 °C (2.186 ± 0.005 mg RE g^−1^), whereas the lowest value was observed in fruit pulp collected during winter and lyophilized (0.021 ± 0.002 mg RE g^−1^), representing an approximately 100-fold difference between the highest and lowest values. These differences were statistically significant among tissues and seasons (Table 2; *p* < 0.05). Among lyophilized samples, leaves exhibited the highest flavonoid concentrations across seasons, differing significantly from fruit pulp and bark (*p* < 0.05). Similarly, for samples oven-dried at 45 °C and 75 °C, the highest concentrations were consistently observed in leaves collected during autumn (Table 2).

Across drying treatments, oven drying at 45 °C produced higher flavonoid concentrations than lyophilization in some samples, particularly in autumn leaves. This may be associated with improved extractability of flavonoids due to partial disruption of plant tissues during moderate thermal drying. In contrast, drying at 75 °C generally resulted in slightly lower concentrations, suggesting possible degradation of thermolabile flavonoids.

### 2.3. Condensed Tannin Content (CTC)

Condensed tannin content was determined in ethanolic extracts of fruit pulp, leaves, and bark of *R. echinocarpa* collected throughout the year and subjected to three drying methods: freeze drying (0.045–0.090 mbar, −85 °C) and oven drying at 45 °C and 75 °C. The CTC was recorded in leaves collected during autumn and dried at 45 °C (0.261 ± 0.003 mg EE g^−1^), whereas the lowest value was observed in bark collected during winter and oven-dried at 45 °C (0.014 ± 0.001 mg EE g^−1^), representing an approximately 19-fold difference between the highest and lowest values. These differences were statistically significant among tissues and seasons (Table 3; *p* < 0.05). Among lyophilized samples, the highest tannin concentrations were observed in leaves collected during spring. Similarly, for samples oven-dried at both 45 °C and 75 °C, the highest concentrations were consistently found in leaves collected during autumn (Table 3).

Across drying treatments, moderate thermal drying (45 °C) preserved or enhanced tannin concentrations in some samples compared with lyophilization, whereas drying at 75 °C generally resulted in lower values, suggesting possible degradation of thermolabile tannin compounds.

### 2.4. HPLC-PDA-MS Analysis

**Figure 1:**
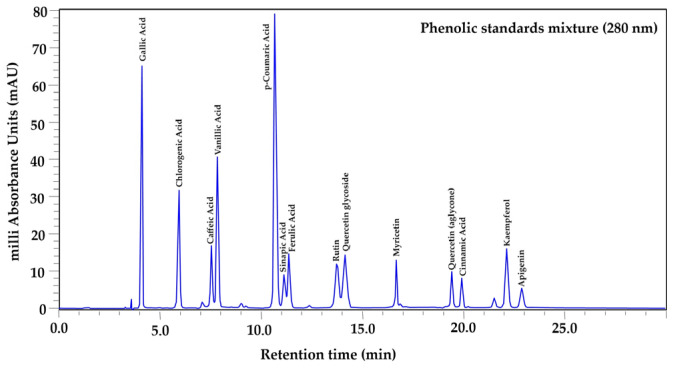
HPLC–PDA chromatogram of a mixture of phenolic reference standards recorded at 280 nm, showing the retention times of the compounds used for identification.

**Figure 2:**
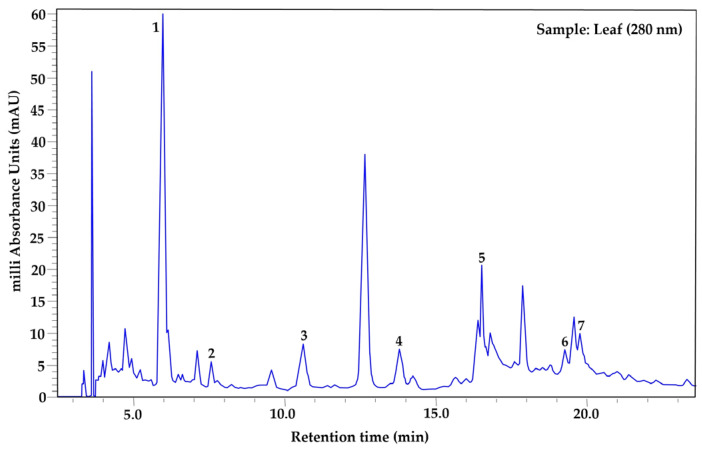
HPLC–PDA chromatogram of *Randia echinocarpa* leaf extract recorded at 280 nm. Peak numbers correspond to the phenolic compounds listed in Table 4.

**Figure 3:**
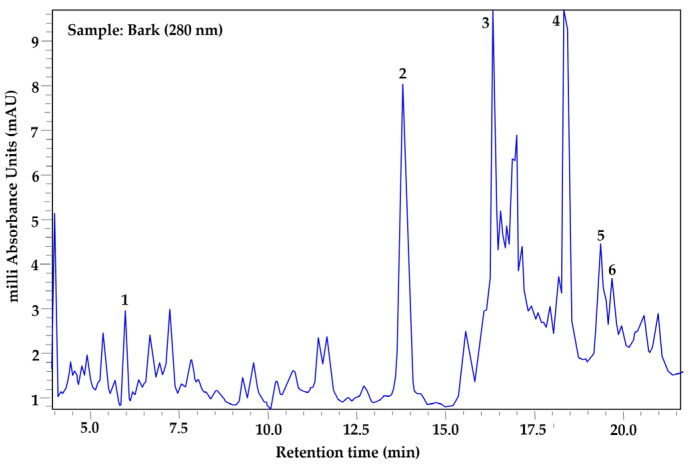
HPLC–PDA chromatogram of *Randia echinocarpa* bark extract recorded at 280 nm. Peak numbers correspond to the phenolic compounds listed in Table 4.

**Figure 4:**
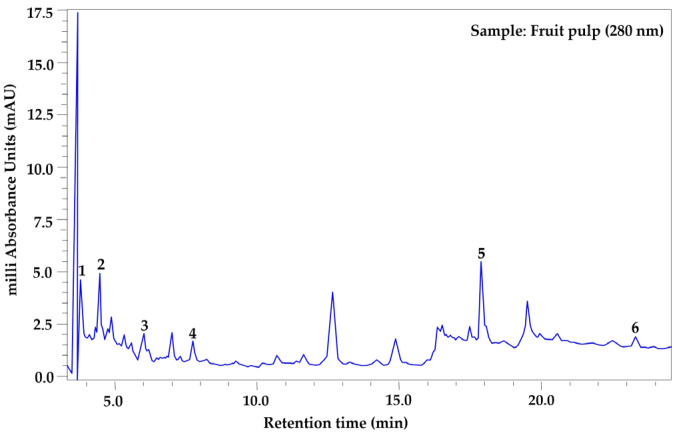
HPLC–PDA chromatogram of *Randia echinocarpa* fruit pulp extract recorded at 280 nm. Peak numbers correspond to the phenolic compounds listed in Table 4.

The phenolic profile was determined exclusively in samples processed using the drying method that best preserved the highest concentrations of total phenols in each tissue (leaf, fruit pulp, and bark), also considering the season of use in traditional medicine: leaf (year-round use), bark (spring–summer), and fruit pulp (autumn–winter). Based on this criterion, leaf samples collected in autumn and oven-dried at 45 °C were analyzed, along with summer bark and winter fruit pulp samples subjected to lyophilization. Prior to extract analysis, a mixture of phenolic standards was analyzed under the same chromatographic conditions (Figure 1). Seven phenolic compounds were identified in the leaf (Figure 2), in the bark six were identified (Figure 3), and in the fruit pulp, six were identified (Figure 4), by HPLC-PDA-MS analysis, as shown in Table 4. Among the phenolic compounds identified, chlorogenic acid, ellagic acid, and catechin exhibited the highest relative abundance, followed by rutin, myricetin, and ferulic acid.

**Table 4:** Phenolic profile in leaf, bark, and fruit pulp of *Randia echinocarpa* by HPLC-PDA-MS.

Tissue	Peak	Compound	RT (min)	λmax (nm)	Observed ion (*m*/*z*; Adduct)	Identification Confidence (MSI)	RA (%)
Leaf	1	Chlorogenic acid	5.95	326	353 [M−H]^−^	Level 1	30.241
	2	Caffeic acid	7.56	297	179 [M−H]^−^	Level 1	1.227
	3	Ferulic acid	10.61	356	193 [M−H]^−^	Level 1	5.335
	4	Rutin	13.79	353	609 [M−H]^−^	Level 1	3.508
	5	Myricetin	16.51	265/347	317 [M−H]^−^	Level 1	2.634
	6	Quercetin	19.25	256/369	301 [M−H]^−^	Level 1	1.096
	7	Cinnamic acid	19.76	320	147 [M−H]^−^	Level 1	1.028
		Unidentified				Level 4	54.931
Bark	1	Chlorogenic acid	5.96	323	353 [M−H]^−^	Level 1	3.1
	2	Rutin	13.81	255/356	609 [M−H]^−^	Level 1	22.636
	3	Myricetin	16.31	254/365	317 [M−H]^−^	Level 1	10.455
	4	Ellagic acid	18.37	250/355	301 [M−H]^−^	Level 2	26.867
	5	Quercetin	19.35	256/370	301 [M−H]^−^	Level 1	2.806
	6	Cinnamic acid	19.69	327	147 [M−H]^−^	Level 1	1.605
		Unidentified				Level 4	32.531
Fruit Pulp	1	Catechin	3.78	286	289 [M−H]^−^	Level 2	10.527
	2	Gallic acid	4.46	295	169 [M−H]^−^	Level 1	3.852
	3	Chlorogenic acid	6.01	331	353 [M−H]^−^	Level 1	4.102
	4	Caffeic acid	7.73	297	179 [M−H]^−^	Level 1	2.01
	5	Myricetin	17.87	255/370	317 [M−H]^−^	Level 1	8.897
	6	Apigenin	23.30	268/336	269 [M−H]^−^	Level 1	1.324
		Unidentified				Level 4	69.288

MSI identification confidence levels according to Sumner et al. [12]: Level 1, compound identified with authentic reference standard (matching RT, UV-Vis, and *m*/*z*) (Figure 1); Level 2, putatively annotated compound based on spectral similarity with databases or literature; Level 3, putatively characterized compound class; Level 4, unknown compound. Relative abundance was estimated from the peak area in the chromatogram at 280 nm and normalized to 100% for each tissue. RT = retention time, RA (%) = relative abundance (%).

### 2.5. DPPH Assay

**Figure 5:**
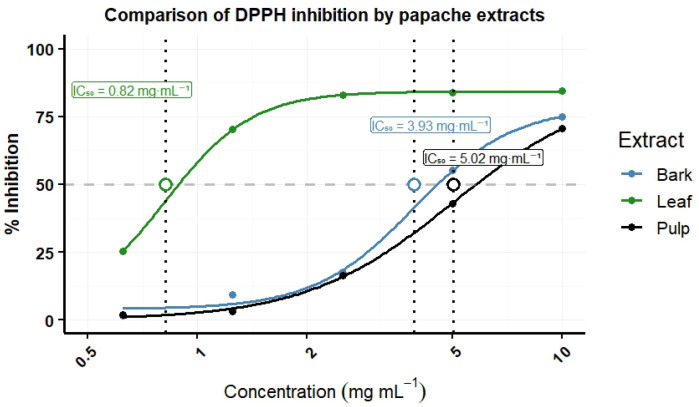
Median inhibition concentration (IC_50_) for the inhibition of DPPH with extracts of fruit pulp, leaf, and bark of *Randia echinocarpa*. The dashed horizontal line represents 50% inhibition, the vertical dashed lines indicate the IC_50_ values, and the circles mark the corresponding points on the fitted curves.

The antioxidant capacity (AC) was determined exclusively in samples processed using the drying method that best preserved the highest concentrations of total phenols in each tissue (leaf, fruit pulp, and bark), also considering the season of use in traditional medicine: leaf (year-round use), bark (spring–summer), and fruit pulp (autumn–winter). Based on this criterion, leaf samples collected in autumn and oven-dried at 45 °C were analyzed, along with summer bark and winter fruit pulp samples subjected to lyophilization (Table 2). The highest radical scavenging activity was observed in leaf extracts, reaching inhibition values above 70% from a concentration of 1.25 mg mL^−1^ and presenting an estimated IC_50_ of 0.82 ± 0.11 mg mL^−1^. In contrast, fruit pulp extracts showed the lowest activity, achieving inhibition values above 50% only at the highest tested concentration (10 mg mL^−1^), with an estimated IC_50_ of 5.02 ± 0.47 mg mL^−1^ (Table 5). Among the evaluated tissues, leaf extracts showed the strongest inhibition of the DPPH radical, followed by bark extracts with an estimated IC_50_ of 3.929 mg mL^−1^, while pulp extracts exhibited lower radical scavenging activity (Table 5; Figure 5).

### 2.6. ABTS Assay

**Figure 6:**
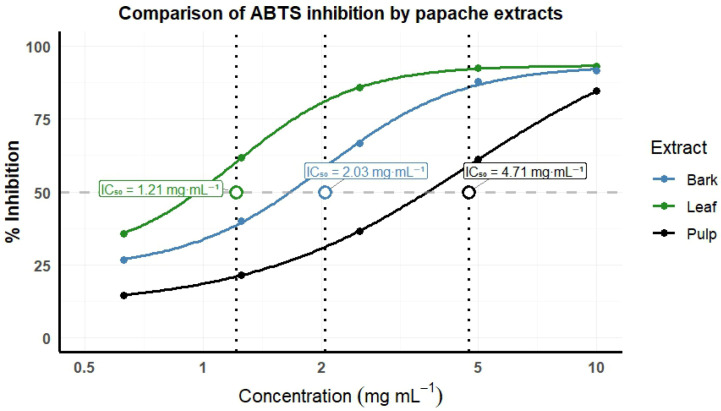
Median inhibition concentration (IC_50_) for the inhibition of ABTS with extracts of fruit pulp, leaf, and bark of *Randia echinocarpa*. The dashed horizontal line represents 50% inhibition, the vertical dashed lines indicate the IC_50_ values, and the circles mark the corresponding points on the fitted curves.

The antioxidant capacity (AC) was determined exclusively in samples processed using the drying method that best preserved the highest concentrations of total phenols in each tissue (leaf, fruit pulp, and bark), also considering the season of use in traditional medicine: leaf (year-round use), bark (spring–summer), and fruit pulp (autumn–winter). Based on this criterion, leaf samples collected in autumn and oven-dried at 45 °C were analyzed, along with summer bark and winter fruit pulp samples subjected to lyophilization (Table 6). Radical scavenging activity in the ABTS assay was observed in leaf extracts, reaching inhibition values above 50% from a concentration of 1.25 mg mL^−1^ and presenting the highest estimated IC_50_, 1.21 ± 0.008 mg mL^−1^. In contrast, fruit pulp extracts showed the lowest activity, achieving inhibition values above 50% from a concentration of 5 mg mL^−1^, with an estimated IC_50_ of 4.71 ± 0.119 mg mL^−1^ (Table 6). Among the evaluated tissues, leaf extracts exhibited the strongest inhibition of the ABTS radical, followed by bark extracts with an estimated IC_50_ of 2.03 ± 0.103 mg mL^−1^, while pulp extracts showed lower radical scavenging activity (Table 6; Figure 6).

## 3. Discussion

### 3.1. Total Phenolic Content (TPC)

Some authors have reported similar concentrations of total phenols to this study, such as those found in methanolic (0.64 mg g^−1^) and aqueous extracts (2.27 mg g^−1^) of the fruit of *Randia echinocarpa* [7]. Moreover, others have reported higher concentrations using aqueous extraction at room temperature and aqueous extraction at boiling temperature (4.84 and 6.28 mg g^−1^, respectively) [8]. Recently, lower concentrations (0.9 mg g^−1^) were reported in dry cell biomass of *R. echinocarpa,* compared to those reported in the present study with extracts of fruit pulp, leaf, and bark [11]. The variations in these concentrations across this and previous investigations could be influenced by harvesting or cultivation season, as well as the specific tissue analyzed, as shown in Table 1. Moderate thermal drying may increase the extractability of compounds due to partial disruption of plant cell structures, facilitating the release of compounds bound to cell wall components. Similar effects have been reported in several plant matrices where drying processes promote the release of bound phenolics and improve extraction efficiency [13,14,15]. Conversely, higher drying temperatures may cause degradation or oxidation of thermolabile phenolic compounds [16,17].

### 3.2. Total Flavonoid Content (TFC)

Flavonoids are natural pigments in plants that protect against damage from oxidative agents such as UV rays, environmental pollution, pathogens, etc., and over 5000 different flavonoids have been identified [18,19,20,21,22]. There are studies limited to detecting only flavonoids presence but studies on the concentration of flavonoids in *R. echinocarpa* are scarce or nonexistent [23,24,25]. Therefore, this study represents the first report on the concentration of these compounds. Confirming their presence in the phenolic profile via HPLC-PDA-MS, five flavonoid compounds were identified: apigenin, quercetin, myricetin, rutin, and catechin (Table 4). In relation to the types of drying, it has been reported that freeze-dried samples can retain more flavonoids compared to those dried in an oven [26], although in the present study a higher concentration was found in leaves collected in autumn and dried at 45 °C, probably because moderate thermal drying can increase the extraction capacity of compounds due to the partial alteration of the structure of plant cells [14,15], in the other seasons and tissues they behaved similarly, but higher than those dried at high temperature (75 °C) because this causes degradation or oxidation of thermolabile flavonoids [17,27].

### 3.3. Condensed Tannin Content (CTC)

These compounds have some antinutritional effects due to their chemical characteristics, causing a bitter taste and inhibiting proteins and digestive enzymes [28]. Similar to flavonoids, studies on concentrations of condensed tannin in *R. echinocarpa* are scarce or nonexistent, limited to their detection only [23,25]. In this study, higher concentrations were found in leaves during the autumn season (possibly derived as a response to increased temperature). However, the concentrations detected were relatively low compared with levels commonly associated with adverse nutritional effects, without making direct safety claims [29]. Similar to total flavonoids, freeze drying is reported to preserve tannins, although moderate temperatures (45 °C) also favor the preservation of phenolic compounds [14,15], while high temperatures (75 °C) cause degradation and oxidation [13,17,30].

### 3.4. HPLC-PDA-MS Analysis

Analyses of the phenolic profile of *Randia echinocarpa* are extremely scarce; a previous investigation focused on dry cell biomass and dry supernatants of this species, identified 11 phenolic compounds, with salicylic acid and chlorogenic acid being the most concentrated [11]. Phenolic profiling has also been conducted on species within the same genus, such as *Randia monantha*, where fruit pulp and seed were analyzed using ultra-high performance liquid chromatography–mass spectrometry (UPLC-MS-MS), revealing 15 phenolic compounds, with chlorogenic acid and rutin being the most concentrated, respectively [31]. These studies reveal a similar trend in phenolic compound diversity within the genus Randia. However, other studies on different plant species have reported greater diversity, such as *Maclura tinctoria* with 33 compounds in bark extracts [32] and *Fouquieria splendens* with 15 phenolic compounds [33]. Differences in the number of phenolic compounds found in this study and those mentioned may be due to specific properties of phenolic compounds in response to biotic and abiotic factors. Additionally, the quantity, type, and concentration of phenolic compounds depend on several factors, including plant tissue and developmental stage [34]. A relatively high proportion of peaks remained classified as “unidentified compounds” (Table 4). This is common in plant metabolomic profiles due to the high chemical complexity of plant extracts and the limited availability of reference standards for many secondary metabolites [35,36]. In the present study, compound identification was based primarily on retention time, UV spectra obtained from the PDA detector, and molecular ions detected by HPLC-PDA-MS analysis. Accordingly, the chromatographic analysis should be considered semi-quantitative, and the reported compounds represent the major phenolic constituents detected under the applied analytical conditions rather than an exhaustive quantitative characterization. Therefore, compounds lacking authentic standards and not present in our internal database were conservatively classified as unidentified. Additionally, the unidentified fraction may be partially explained by the possible co-elution of compounds and the presence of low-abundance metabolites.

### 3.5. DPPH Assay

The lowest IC_50_ value in the DPPH assay was observed in ethanolic extracts of *Randia echinocarpa* leaves (IC_50_ = 0.82 mg mL^−1^), followed by bark and fruit pulp extracts, the latter showing the lowest radical scavenging activity (IC_50_ = 5.02 mg mL^−1^) (Figure 5; Table 5). These values differed from those reported for hydroalcoholic extracts of *Caesalpinia spinosa* pods (IC_50_ = 3.2 μg mL^−1^) [37], but were comparable to those observed in acetic extracts of *Oxalis tuberosa* peel (IC_50_ = 5.37 mg mL^−1^) Likewise, leaf extracts of *R. echinocarpa* showed lower IC_50_ values than those reported for methanolic extracts of *Randia monantha* pulp (IC_50_ = 1 mg mL^−1^) [31]. These differences may be associated with variability in the composition and concentration of antioxidant metabolites among plant tissues, as well as with the affinity of the extraction solvent, which influences the recovery of phenolic compounds [38,39]. Additionally, post-harvest processing methods such as drying can influence the preservation of bioactive compounds and their final concentration in plant extracts [40,41]. The absence of reference antioxidant standard limits direct comparison of antioxidant potency with other studies; therefore, the DPPH assay results were interpreted primarily as a comparative evaluation among *R. echinocarpa* tissues. It should be noted that antioxidant activity was evaluated only in a selected subset of samples chosen based on phenolic content and ethnobotanical relevance; therefore, these results do not represent the entire dataset.

### 3.6. ABTS Assay

The lowest IC_50_ value in the ABTS assay was observed in *Randia echinocarpa* leaf extracts (IC_50_ = 1.21 mg mL^−1^), followed by bark (IC_50_ = 2.03 mg mL^−1^) and fruit pulp (IC_50_ = 4.71 mg mL^−1^), indicating differences in radical scavenging activity among plant tissues (Figure 6; Table 6). A similar pattern has been reported in *Cassine glauca*, where leaves exhibited higher concentrations of phenolic compounds and lower IC_50_ values compared with bark [42]. Likewise, in *Chionanthus pubescens*, leaf extracts showed lower IC_50_ values than other plant tissues [43]. These differences may be associated with the accumulation of defensive secondary metabolites, such as flavonoids and phenolic acids, in leaves, whereas tissues such as fruit pulp tend to accumulate higher proportions of sugars and pigments [44]. Overall, the results indicate that plant tissue influences radical scavenging activity, with leaf extracts showing lower IC_50_ values under the evaluated experimental conditions. Regarding the drying method, previous studies have demonstrated that freeze drying and moderate-temperature drying can preserve antioxidant compounds more effectively than high-temperature drying [40]. In agreement with this, drying at 45 °C better preserved antioxidant activity, whereas drying at 75 °C was associated with greater degradation of phenolic compounds and a consequent reduction in antioxidant capacity [15,16,17,41]. It should be noted that antioxidant activity was evaluated only in a selected subset of samples chosen based on phenolic content and ethnobotanical relevance; therefore, these results do not represent the entire dataset.

### 3.7. Impact of Environmental Factors on Bioactive Compounds (Phenolic Profile, Concentration, and Antioxidant Capacity)

Environmental conditions can influence the synthesis and accumulation of secondary metabolites in medicinal plants. Factors such as altitude, temperature regimes, and precipitation patterns can modulate biosynthetic pathways involved in the production of phenolic compounds, flavonoids, and tannins, which are frequently associated with antioxidant activity [45,46,47]. In tropical deciduous and subdeciduous forests, seasonal drought and high solar radiation can promote the accumulation of metabolites involved in antioxidant defense and protection against abiotic stress [48]. Similarly, higher altitudes may promote increased concentrations of phenolic compounds and flavonoids due to greater ultraviolet radiation and wider thermal amplitudes [49].

Seasonal variation in phenolic composition and antioxidant activity has been reported in several medicinal plants. For example, *Cistus creticus* and Mediterranean aromatic species, such as sage (*Salvia officinalis* L.), spearmint (*Mentha spicata* L.), and *Sideritis perfoliata subsp. perfoliata* L., show seasonal fluctuations in phenolic content and antioxidant activity, with higher values generally reported during winter [47,50]. A similar seasonal pattern was observed in the present study, where leaf samples collected during autumn and winter showed higher phenolic concentrations and lower IC_50_ values. This trend is consistent with reports suggesting that environmental stress may contribute to polyphenol accumulation, compounds known for their antioxidant and antimicrobial properties. [18,19,51,52,53,54].

Plants represent an important source of bioactive metabolites. Phenolic compounds such as chlorogenic acid, rutin, and ellagic acid (Table 4) may vary according to seasonal conditions [7,24,55]; for example, chlorogenic acid peaks in late spring in *Helichrysum aureonitens*, whereas ellagic acid and rutin reach their highest levels in late summer in walnut leaves (*Juglans regia*) [56,57]. Periods of higher polyphenol abundance have frequently been associated with increased antioxidant activity [51]. In *Cistus creticus*, leaf extracts collected in winter showed the lowest IC_50_ values in DPPH assays, while summer samples were more effective against ABTS radicals. Similarly, in the present study, leaf extracts showed lower IC_50_ values than bark and fruit pulp in both assays, suggesting that seasonal variation in phenolic composition may influence antioxidant activity [47,51].

Environmental variables such as temperature, precipitation, and solar radiation were not directly measured in this study; therefore, their potential influence on bioactive compound accumulation is interpreted based on patterns reported in the literature.

## 4. Materials and Methods

### 4.1. Sample Collection

**Figure 7:**
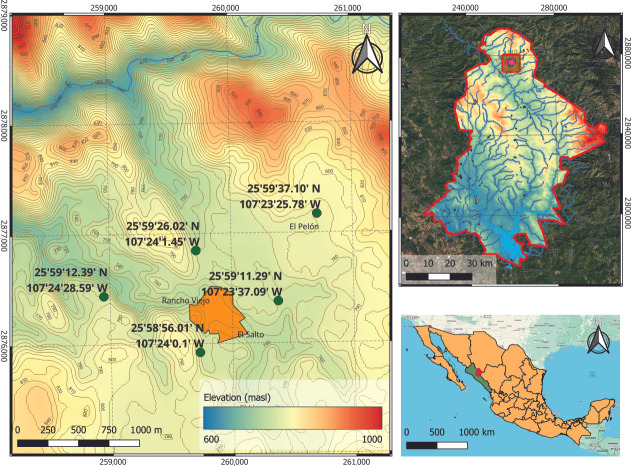
Collection areas surrounding the community of Rancho Viejo in the municipality of Badiraguato, Sinaloa, Mexico (green circles represent the place of the collected sample).

Four sampling campaigns were conducted throughout an annual cycle during 2023 to collect bark, leaves, and fruit pulp from wild *Randia echinocarpa* plants. Sampling was performed in five locations surrounding the Rancho Viejo community in the municipality of Badiraguato, Sinaloa, Mexico (25°59′14″ N, 107°24′04″ W; 743 m.a.s.l.) (Figure 7).

At each sampling event, plant material was collected from ten reproductively mature individuals (reproductive maturity is estimated to occur after 2 to 5 years) per sampling site, across five sampling locations, resulting in a total of 50 individuals sampled per season and 200 individuals sampled throughout the study period (*n* = 200). This sampling strategy was intended to capture spatial and seasonal variability in phenolic composition in natural populations of *Randia echinocarpa*. Healthy tissues were selected from each plant. Stems with abundant leaves were collected, obtaining approximately 250 g of fresh material per sampling point, which were stored in 50 × 20 cm paper bags. Additionally, approximately 20 mature fruits (≈2.5 kg) were collected and stored in 50 × 100 cm black bags for subsequent processing.

#### Plant Identification

Species identification was performed with the assistance of the botanist José Saturnino Díaz, PhD. A voucher specimen of *Randia echinocarpa* Moc. & Sessé ex DC. was deposited in the Herbarium of the Faculty of Zootechnics and Ecology of the Autonomous University of Chihuahua (UACH-HER) under accession number JSD0031.

### 4.2. Sample Processing

**Figure 8:**
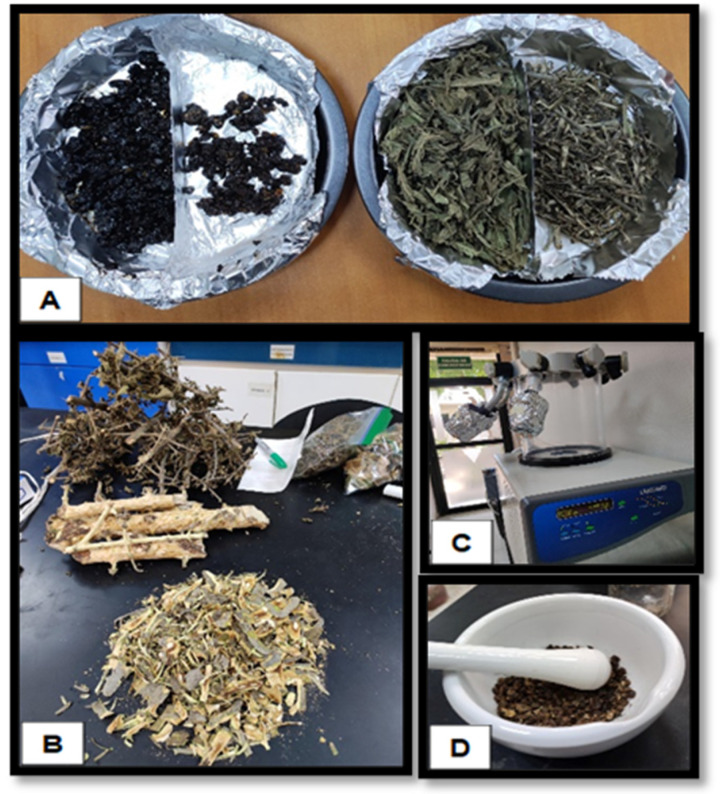
Plant tissue processing. Preparation of fruit pulp, leaf, and bark for oven drying (**A**), separation of plant tissues (**B**), lyophilization of samples (**C**), and pulverization of dried material (**D**).

The tissues were manually separated, taking care to avoid exposure to light to prevent oxidation. Plant material collected from the different individuals within each sampling site was pooled and homogenized to obtain representative samples for subsequent phytochemical analysis. The plant tissues (leaves, bark, and fruit pulp) were manually separated prior to drying. For lyophilization, each tissue was placed in glass flasks suitable for the freeze-dryer (FreeZone 2.5 L, Labconco, Kansas City, MO, USA) and pre-frozen at −80 °C for 24 h. Samples were then freeze-dried under the following operating conditions: 0.045–0.090 mbar and −85 °C. For oven drying, the separated tissues were placed on aluminum trays and dried in a laboratory oven (Yamato, IC103CW, Tokyo, Japan) at 45 °C or 75 °C. Drying times varied depending on tissue type and moisture content, ranging from approximately 1 to 7 days until constant weight was achieved (Figure 8). Subsequently, they were pulverized using a household blender (Toastmaster, FL, USA). The pulverized samples were reduced to a particle size of less than 1 mm using a hammer mill (LUZ-1143, Luzeren, Shenzhen, China). The samples were then stored in aluminum-covered jars and kept at ultra-low temperatures at −80 °C until use. During the drying process, the water content was estimated, and it was found that the leaf had 60%, the bark had 25%, and the fruit pulp had 63%.

### 4.3. Extraction of Phenolic Compounds by Ethanol Extraction

One gram of each sample of dried tissue was taken and macerated in 10 mL of 50% ethanol–water (*v*/*v*) (Baker, Phillipsburg, NJ, USA) for 24 h at room temperature, in the dark (all extractions were performed in triplicate). The resulting extracts were centrifuged at 4000× *g* for 15 min (Eppendorf Centrifuge 5804, Hamburg, Germany), and the supernatants were recovered in capped tubes protected from light with aluminum foil and used to determine TPC, TFC, CTC, and AC [58]. All results were expressed on a dry weight basis since plant materials were dried prior to extraction. The analytical procedures were conducted following previously reported protocols commonly used for phenolic and antioxidant analyses. All spectrophotometric analyses were performed in triplicate to ensure analytical reproducibility.

### 4.4. Determination of Phenolic Profile and Antioxidant Capacity

#### 4.4.1. HPLC-PDA-MS Analysis

The phenolic profile was determined by direct comparison of the mass spectra using high-performance liquid chromatography–photodiode array detector–mass spectrometry (HPLC-PDA-MS). Sample selection was based on the drying method that best preserved total phenolic concentrations in each tissue (leaf, bark, and fruit pulp). In addition, the season in which each tissue is traditionally used in local medicinal practices was considered: leaf (year-round), bark (spring–summer), and fruit pulp (autumn–winter).

The extraction was carried out as described in Section 4.3 and subsequently dried by rotoevaporation and resuspended in 3 mL of methanol HPLC grade (J.T. Baker, Phillipsburg, NJ, USA) (0.021 g).

The HPLC profile of phenolics in the methanolic crude extract was established with an LC-MS 2020 system (Shimadzu, Kyoto, Japan) coupled to a photodiode array detector (SPD-M20A, UV-Vis, Shimadzu, Kyoto, Japan) and a single-quadrupole mass spectrometer with an electrospray ionization source (ESI) (LC-MS-2020, Shimadzu, Kyoto, Japan). The system included a CBM-20A controller (Shimadzu, Kyoto, Japan), two binary pumps (LC-20AD, Shimadzu, Kyoto, Japan), a degasser (DGU-20A5R, degasser (DGU-20A5R), an autosampler (SIL-20AC, Shimadzu, Kyoto, Japan), and a column oven (CTO-20A, Shimadzu, Kyoto, Japan). Separation was carried out using an analytical RP-18 reversed-phase column (Luna 100, 250 × 4.0 mm, 5 µm) from Phenomenex (Torrance, CA, USA), maintained at 30 °C in a column oven. Data acquisition and processing were performed using LabSolutions software version 5.0 (Shimadzu, Kyoto, Japan). A binary mobile phase consisting of Milli-Q water with 0.2% (*v*/*v*) formic acid (phase A) and methanol with 0.2% (*v*/*v*) formic acid (phase B), both HPLC grade (J.T. Baker, Phillipsburg, NJ, USA), was used. Elution was performed using a linear gradient of solvent B as follows: 0.01–1 min, 30% B; 1–3 min, 33% B; 3–7 min, 37% B; 7–13 min, 40% B; 13–16 min, 55% B; 16–22 min, 60% B; 22–25 min, 45% B; 25–27 min, 30% B. These conditions were maintained until a total time of 27 min, including column re-equilibration.

The flow rate was kept constant at 1.0 mL min^−1^, and the injection volume was 20 µL for HPLC-PDA and 10 µL for HPLC-MS. UV-Vis spectra were recorded with the PDA detector in the 200–800 nm range, monitoring chromatograms at 280 and 320 nm. The mass spectrometer (LCMS-2020, Shimadzu, Kyoto, Japan) operated with an electrospray ionization (ESI) source in both positive and negative ionization modes. The capillary voltage was set at 4.5 kV. The interface temperature was maintained at 350 °C and the desolvation line temperature at 250 °C. Nitrogen was used as a nebulizing and drying gas. The nebulizing gas flow was set at 1.5 L min^−1^ and the drying gas flow at 15 L min^−1^. Mass spectra were acquired with a scan range of *m*/*z* 50–1000.

The chromatographic peaks were identified by comparing retention times, UV spectra obtained by the PDA detector and *m*/*z* values obtained by ESI-MS, compared with pure standards (i.e., gallic, chlorogenic, caffeic, vanillic, p-coumaric, sinapic, ferulic, and cinnamic acids and the flavonoids (+) rutin hydrate, quercetin dihydrate, glycosylated quercetin, kaempferol, apigenin, and myricetin). For the standards, individual stock solutions (≈1000 mg L^−1^) of each standard were prepared by weighing the appropriate amount, correcting for purity and hydration state, and dissolving in methanol. The solutions were filtered through 0.22 µm membranes and stored in amber vials at −20; from these stocks, intermediate mixtures (≈100 mg L^−1^ each) are prepared, one for phenolic acids and another for flavonoids, to be analyzed by dilution from these mixtures (acids: 0.5–50 mg L^−1^; flavonoids: 0.25–25 mg L^−1^). Each sample was injected in triplicate. Data acquisition and processing were performed using LabSolutions software version 5.0 (Shimadzu, Kyoto, Japan [58,59,60]. Because compound identification was based primarily on retention times, UV spectra, and molecular ions obtained by single-quadrupole MS, the chromatographic analysis should be considered semi-quantitative. Results are reported as relative abundances rather than absolute concentrations, as the objective of the analysis was qualitative and semi-quantitative profiling.

#### 4.4.2. Total Phenolic Content (TPC)

The total phenolic content was determined using the Folin–Ciocalteu method adapted for microplate assays. In a microplate, 140 μL of distilled water, 10 μL of the sample or standard, and 10 μL of Folin’s reagent were mixed and allowed to stand for 6 min in the dark. Then, 40 μL of 7.5% (*w*/*v*) sodium carbonate solution was added, and the reaction mixture was mixed and incubated at 45 °C for 15 min in the dark. The absorbance was read at 760 nm using a spectrophotometer (Multiskan GO, Thermo Scientific, Vantaa, Finland). Gallic acid was used as the standard at different concentrations, and the results are expressed as milligrams of gallic acid equivalents per gram of dry tissue (mg GAE g^−1^) [61].

#### 4.4.3. Total Flavonoid Content (TFC)

The determination of total flavonoid content required mixing 100 μL of the ethanolic extract with 130 μL of distilled water and 20 μL of a 1% (*w*/*v*) solution of 2-aminoethyl diphenylborinate in a 96-well microplate. The absorbance of the solution was immediately read at 404 nm using a spectrophotometer (Multiskan GO, Thermo Scientific, Vantaa, Finland). Rutin at different concentrations was used as the standard; in this case, 50 μL of rutin was mixed with 180 μL of distilled water and 20 μL of 2-aminoethyl diphenylborinate. The flavonoid content is expressed as milligrams of rutin equivalents per gram of dry tissue (mg RE g^−1^) [62].

#### 4.4.4. Condensed Tannin Content (CTC)

The determination of condensed tannins was performed by preparing a 1% (*w*/*v*) vanillin solution in 100% methanol on the day of analysis and mixing it with an 8% (*v*/*v*) HCl solution (prepared in 100% methanol) in a 1:1 ratio to obtain a 0.5% (*w*/*v*) vanillin reagent solution. Epicatechin at different concentrations was used as the standard. Then, 40 μL of extract or standard was mixed with 200 μL of 0.5% (*w*/*v*) vanillin reagent solution. The reaction mixture was incubated at 30 °C for 20 min and the absorbance was read at 500 nm using a spectrophotometer (Multiskan GO, Thermo Scientific, Vantaa, Finland). The condensed tannin content is expressed as milligrams of epicatechin equivalents per gram of dry tissue (mg EE g^−1^) [63].

#### 4.4.5. DPPH Assay

Antioxidant capacity assays were performed on a selected subset of samples rather than on the entire dataset. Sample selection was based on the drying method that best preserved total phenolic concentrations in each tissue (leaf, bark, and fruit pulp). In addition, the season in which each tissue is traditionally used in local medicinal practices was considered: leaf (year-round), bark (spring–summer), and fruit pulp (autumn–winter).

The DPPH assay was adapted to a microplate [64]. The 2,2-diphenyl-1-picrylhydrazyl (DPPH) radical was prepared at 150 µM in 80% (*v*/*v*) methanol. A 1.5 mg amount of DPPH was used with 20 mL of 80% methanol; subsequently, it was brought to a volume of 25 mL. The solution was sonicated for 15 min. In a 96-well plate, the reading of the freshly prepared DPPH was taken and adjusted to 0.8 absorbance with 80% methanol. Five concentrations of the extracts (leaf, bark, and pulp) were prepared by serial dilution (10, 5, 2.5, 1.25, and 0.625 mg mL^−^^1^). The plate was prepared with 20 µL of the sample in its respective independent wells, and 200 µL of DPPH (150 µM) was added. The absorbance at 515 nm was recorded after 30 min in a spectrophotometer (Thermo Scientific, Multiskan GO, Vantaa, Finland). The inhibition percentages were calculated using the following formula:

$$\mathrm{Inhibition}\;(\%)\;=\;\left(\frac{A_{control}-A_{sample}}{A_{control}}\right)\times 100$$

where:

-A_Control_ = initial absorbance of DPPH;

-A_sample_ = absorbance of the concentrations/samples to be tested.

Subsequently, a nonlinear regression was used by fitting an inhibition curve based on a four-parameter logistic (4PL) model using RStudio software 2026.01.1 with the drc and ggplot2 packages [65,66]:

$$Y(X)\;=\;\frac{A-D}{1+{\left(\frac{X}{C}\right)}^{B}}+D$$

where:

-Y: % inhibition;

-X: extract concentration (mg mL^−1^);

-A: upper asymptote value (maximum inhibition);

-D: lower asymptote value (minimum inhibition);

-B: slope or “Hill Slope”;

-C: IC_50_ (concentration for 50% inhibition).

The fitted curve was interpolated, and the results were reported as mean inhibitory concentration (IC_50_), with 95% confidence intervals calculated using the delta method [66]. The assay was performed to compare the radical scavenging activity among the evaluated tissues of *R. echinocarpa* under the same conditions.

#### 4.4.6. ABTS Assay

Antioxidant capacity assays were performed on a selected subset of samples rather than on the entire dataset. Sample selection was based on the drying method that best preserved total phenolic concentrations in each tissue (leaf, bark, and fruit pulp). In addition, the season in which each tissue is traditionally used in local medicinal practices was considered: leaf (year-round), bark (spring–summer), and fruit pulp (autumn–winter).

The ABTS•^+^ assay was adapted to a microplate [67]. It consisted of generating the radical from the reaction between 2,2′-azino-bis (3-ethylbenzothiazoline-6-sulfonic acid) (ABTS) (7 mM) and potassium persulfate (2.45 mM), incubated for 12–16 h in the dark. For this, a stock solution was prepared by mixing 96 mg of ABTS and 16.55 mg of potassium persulfate in a 25 mL volumetric flask, to which 20 mL of distilled water was added, and then the mixture was sonicated for 10 min and calibrated. It was protected from light and stored at −20 °C. Subsequently, a working ABTS solution was prepared by diluting 1 mL of the stock solution in 50 mL of distilled water. Five concentrations of the extracts (leaf, bark, and pulp) were prepared by serial dilution (10, 5, 2.5, 1.25, and 0.625 mg mL^−1^). In a 96-well microplate, the freshly prepared ABTS was read and adjusted to 0.773 absorbance with 80% methanol at 735 nm. A 20 µL amount of both the extract to be evaluated and the blank (200 µL of the ABTS solution) was added to the same microplate. The reading was taken immediately after loading the microplate at 735 nm in a spectrophotometer (Thermo Scientific, Multiskan GO, Vantaa, Finland). The inhibition percentages were calculated with the following formula:

$$\mathrm{Inhibition}\;(\%)\;=\;\left(\frac{A_{control}-A_{sample}}{A_{control}}\right)\times 100$$

where:

-A_Control_ = initial absorbance of ABTS;

-A_sample_ = absorbance of the concentrations/samples to be tested.

Subsequently, a nonlinear regression was used by fitting an inhibition curve based on a four-parameter logistic (4PL) model using RStudio software 2026.01.1 with the drc and ggplot2 packages [65,66]:

$$Y(X)=\frac{A-D}{1+{\left(\frac{X}{C}\right)}^{B}}+D$$

where:

-Y: % inhibition;

-X: extract concentration (mg mL^−1^);

-A: upper asymptote value (maximum inhibition);

-D: lower asymptote value (minimum inhibition);

-B: slope or “Hill Slope”;

-C: IC_50_ (concentration for 50% inhibition).

The fitted curve was interpolated, and the results were reported as mean inhibitory concentration (IC_50_), with 95% confidence intervals calculated using the delta method [66]. The assay was performed to compare the radical scavenging activity among the evaluated tissues of *R. echinocarpa* under the same conditions.

### 4.5. Statistical Analysis

All experiments were performed at least in triplicate. The results are presented as mean values ± standard error (Mean ± SE). The normality of the data was evaluated using the Kolmogorov–Smirnov test. Because plant material from multiple individuals was pooled prior to extraction to obtain composite samples for each tissue–season–drying combination, the triplicate measurements correspond to analytical replicates rather than independent biological replicates. Therefore, the dataset does not represent a fully factorial design. For this reason, statistical comparisons were performed using one-way ANOVA (*p* ≤ 0.05) to evaluate differences among individual tissue–season–drying treatment combinations. When significant differences were detected, Tukey’s test was applied for multiple comparisons. In this context, the statistical analysis was intended to identify differences among specific treatment combinations, not to infer factorial interaction effects. Different letters indicate significant differences (*p* ≤ 0.05).

## 5. Conclusions

HPLC–PDA–MS analysis revealed the presence of several phenolic compounds in *Randia echinocarpa*, including chlorogenic acid, ellagic acid, rutin, caffeic acid, myricetin, and catechin. The results showed that phenolic accumulation varied according to plant tissue, season, and drying method. Leaves generally presented the highest concentrations of phenolic compounds, whereas bark tended to exhibit lower concentrations. Moderate thermal drying (45 °C) frequently preserved phenolic compounds more effectively than higher drying temperatures, suggesting improved extractability for these compounds under moderate drying conditions.

This study also reports for the first time the presence of flavonoids and condensed tannins in *R. echinocarpa*. The results indicate that plant tissue, seasonal variation, and post-harvest processing influence the phenolic composition of this species and its antioxidant capacity. Leaf extracts consistently showed the strongest antioxidant activity in both DPPH and ABTS assays, which may be associated with the higher phenolic concentrations observed in this tissue.

Overall, these findings contribute to the phytochemical characterization of *R. echinocarpa*, a medicinal plant traditionally used in northwestern Mexico. The study provides new information on the distribution of phenolic compounds across tissues and seasons and highlights the importance of post-harvest processing in preserving bioactive compounds. These results provide a basis for future studies aimed at further characterizing the bioactive compounds present in this species and evaluating their biological activity and bioavailability.

## Figures and Tables

**Table 1:** TPC in ethanolic extracts of fruit pulp, leaf, and bark of *R. echinocarpa* collected over an annual cycle and dried via lyophilization and in an oven at 45 °C and 75 °C.

		Lyophilized	Oven at 45 °C	Oven at 75 °C
Tissue	Season	Concentration (mg GAE g^−1^)		
Fruit pulp	Autumn	0.788 ± 0.065 ^aA^	1.287 ± 0.147 ^cB^	1.374 ± 0.109 ^cB^
Fruit pulp	Winter	2.546 ± 0.066 ^bA^	1.467 ± 0.063 ^acB^	0.758 ± 0.049 ^dC^
Leaf	Winter	2.448 ± 0.023 ^bA^	1.988 ± 0.054 ^bB^	1.751 ± 0.114 ^abC^
Leaf	Autumn	2.617 ± 0.048 ^bA^	2.770 ± 0.011 ^fB^	2.683 ± 0.036 ^eA^
Leaf	Spring	2.531 ± 0.047 ^bA^	1.862 ± 0.197 ^abB^	1.782 ± 0.088 ^abB^
Leaf	Summer	2.427 ± 0.043 ^bA^	1.674 ± 0.197 ^abB^	1.662 ± 0.088 ^abB^
Bark	Winter	0.736 ± 0.007 ^aA^	0.437 ± 0.009 ^dB^	0.511 ± 0.059 ^dB^
Bark	Autumn	1.296 ± 0.085 ^cA^	0.872 ± 0.017 ^eB^	1.812 ± 0.099 ^bC^
Bark	Spring	1.850 ± 0.077 ^dA^	1.779 ± 0.125 ^abA^	1.558 ± 0.082 ^abcB^
Bark	Summer	2.539 ± 0.100 ^bA^	1.813 ± 0.108 ^abB^	1.520 ± 0.002 ^acB^

Data are presented as mean ± standard error (*n* = 3). Different capital letters show significant differences between rows (*p* ≤ 0.05). Different lowercase letters show significant differences between columns (*p* ≤ 0.05).

**Table 2:** TFC in ethanolic extracts of fruit pulp, leaf, and bark of *R. echinocarpa* collected over an annual cycle and dried via lyophilization and in oven at 45 °C and 75 °C.

		Lyophilized	Oven at 45 °C	Oven at 75 °C
Tissue	Season	Concentration (mg RE g^−1^)		
Fruit pulp	Autumn	0.090 ± 0.004 ^bA^	0.101 ± 0.001 ^aB^	0.125 ± 0.002 ^aC^
Fruit pulp	Winter	0.021 ± 0.002 ^bA^	0.205 ± 0.004 ^aB^	0.114 ± 0.016 ^aC^
Leaf	Winter	1.287 ± 0.061 ^aA^	1.286 ± 0.109 ^bA^	0.996 ± 0.001 ^bcB^
Leaf	Autumn	1.309 ± 0.075 ^aA^	2.186 ± 0.005 ^eB^	1.247 ± 0.179 ^cA^
Leaf	Spring	1.478 ± 0.104 ^aA^	1.201 ± 0.249 ^bA^	1.083 ± 0.082 ^bcB^
Leaf	Summer	1.346 ± 0.064 ^aA^	1.012 ± 0.059 ^bB^	1.028 ± 0.072 ^bcB^
Bark	Winter	0.386 ± 0.009 ^cA^	0.209 ± 0.015 ^aB^	0.222 ± 0.005 ^adB^
Bark	Autumn	0.455 ± 0.009 ^cdA^	0.364 ± 0.016 ^acB^	0.468 ± 0.028 ^deA^
Bark	Spring	0.630 ± 0.0003 ^dA^	0.683 ± 0.009 ^cdA^	0.664 ± 0.053 ^efA^
Bark	Summer	1.018 ± 0.0002 ^eA^	0.959 ± 0.0002 ^bdB^	0.848 ± 0.0002 ^bfC^

Data are presented as mean ± standard error (*n* = 3). Different capital letters show significant differences between rows (*p* ≤ 0.05). Different lowercase letters show significant differences between columns (*p* ≤ 0.05).

**Table 3:** CTC in ethanolic extracts of fruit pulp, leaf, and bark of *R. echinocarpa* collected over an annual cycle and dried via lyophilization and in oven at 45 °C and 75 °C.

		Lyophilized	Oven at 45 °C	Oven at 75 °C
Tissue	Season	Concentration (mg EE g^−1^)		
Fruit pulp	Autumn	0.037 ± 0.001 ^aA^	0.037 ± 0.002 ^cA^	0.046 ± 0.002 ^bB^
Fruit pulp	Winter	0.042 ± 0.003 ^aA^	0.078 ± 0.002 ^aB^	0.058 ± 0.0001 ^cC^
Leaf	Winter	0.097 ± 0.001 ^cA^	0.067 ± 0.002 ^eB^	0.070 ± 0.002 ^adB^
Leaf	Autumn	0.111 ± 0.001 ^cA^	0.261 ± 0.003 ^fB^	0.094 ± 0.001 ^fC^
Leaf	Spring	0.127 ± 0.007 ^dA^	0.079 ± 0.003 ^aB^	0.075 ± 0.001 ^aB^
Leaf	Summer	0.103 ± 0.002 ^cA^	0.053 ± 0.003 ^aB^	0.073 ± 0.003 ^aC^
Bark	Winter	0.029 ± 0.001 ^aA^	0.014 ± 0.001 ^bB^	0.017 ± 0.001 ^eB^
Bark	Autumn	0.042 ± 0.001 ^aA^	0.045 ± 0.001 ^dA^	0.066 ± 0.002 ^cdB^
Bark	Spring	0.059 ± 0.008 ^cA^	0.077 ± 0.002 ^aB^	0.079 ± 0.006 ^aC^
Bark	Summer	0.097 ± 0.003 ^bA^	0.080 ± 0.0003 ^aB^	0.038 ± 0.001 ^bC^

Data are presented as mean ± standard error (*n* = 3). Different capital letters show significant differences between rows (*p* ≤ 0.05). Different lowercase letters show significant differences between columns (*p* ≤ 0.05).

**Table 5:** Antioxidant activity of *Randia echinocarpa* leaf, bark, and fruit pulp extracts against DPPH.

Conc.	Inhibition (%)		
mg mL^−1^	Leaf	Bark	Fruit pulp
10	84.61	74.90	70.57
5	83.92	55.38	42.91
2.5	83.06	17.44	16.50
1.25	70.21	9.19	3.13
0.625	25.49	1.73	1.83
Estimated IC_50_ (mg mL^−1^ )	0.82 ± 0.11	3.93 ± 0.41	5.02 ± 0.47
95% CI ( mg mL^−1^ )	(0.59–2.23)	(1.34–9.20)	(0.96–11.01)
R^2^	0.999	0.996	0.999

IC_50_ values were estimated using a four-parameter logistic (4PL) regression model. Confidence intervals (95%) were calculated using the delta method. R^2^ represents the goodness-of-fit of the nonlinear regression model.

**Table 6:** Antioxidant activity of *Randia echinocarpa* leaf, bark, and fruit pulp extracts against ABTS.

Conc.	Inhibition (%)		
mg mL^−1^	Leaf	Bark	Fruit pulp
10	93.16	91.52	84.53
5	92.36	87.77	61.27
2.5	85.88	66.66	36.62
1.25	61.73	40.07	21.62
0.625	35.79	26.83	14.62
Estimated IC_50_ (mg mL^−1^ )	1.21 ± 0.008	2.03 ± 0.103	4.71 ± 0.119
95% CI ( mg mL^−1^ )	(1.10–1.32)	(0.72–3.34)	(3.20–6.23)
R^2^	0.999	0.999	0.999

IC_50_ values were estimated using a four-parameter logistic (4PL) regression model. Confidence intervals (95%) were calculated using the delta method. R^2^ represents the goodness-of-fit of the nonlinear regression model.

## Data Availability

Data is contained within this article.
